# Hierarchical traits distances explain grassland Fabaceae species' ecological niches distances

**DOI:** 10.3389/fpls.2015.00063

**Published:** 2015-02-17

**Authors:** Florian Fort, Claire Jouany, Pablo Cruz

**Affiliations:** ^1^Montpellier SupAgro, Centre d'Ecologie Fonctionnelle et Evolutive (Unité Mixte de Recherche 5175)Montpellier, France; ^2^Institut National de la Recherche Agronomique, Unité Mixte de Recherche 1248 Agroécologie Innovation et TerritoireCastanet-Tolosan, France; ^3^National Polytechnic Institute of Toulouse (INPT), Unité Mixte de Recherche AGIR1248, Université de ToulouseToulouse, France

**Keywords:** Ellenberg indicator, mycorrhizal rate, nodule biomass, root depth, root functional trait, specific root length

## Abstract

*Fabaceae* species play a key role in ecosystem functioning through their capacity to fix atmospheric nitrogen via their symbiosis with *Rhizobium* bacteria. To increase benefits of using *Fabaceae* in agricultural systems, it is necessary to find ways to evaluate species or genotypes having potential adaptations to sub-optimal growth conditions. We evaluated the relevance of phylogenetic distance, absolute trait distance and hierarchical trait distance for comparing the adaptation of 13 grassland *Fabaceae* species to different habitats, i.e., ecological niches. We measured a wide range of functional traits (root traits, leaf traits, and whole plant traits) in these species. Species phylogenetic and ecological distances were assessed from a species-level phylogenetic tree and species' ecological indicator values, respectively. We demonstrated that differences in ecological niches between grassland *Fabaceae* species were related more to their hierarchical trait distances than to their phylogenetic distances. We showed that grassland *Fabaceae* functional traits tend to converge among species with the same ecological requirements. Species with acquisitive root strategies (thin roots, shallow root systems) are competitive species adapted to non-stressful meadows, while conservative ones (coarse roots, deep root systems) are able to tolerate stressful continental climates. In contrast, acquisitive species appeared to be able to tolerate low soil-P availability, while conservative ones need high P availability. Finally we highlight that traits converge along the ecological gradient, providing the assumption that species with similar root-trait values are better able to coexist, regardless of their phylogenetic distance.

## Introduction

Reducing the use of fertilizers and their economic and ecological costs is one of the greatest agronomic and environmental challenges of the twenty-first century. To reach this goal it is necessary to select species or genotypes according to their adaptation to sub-optimal growth conditions (Lynch, 2007; Richardson et al., 2011). For example, *Fabaceae's* ability to fix atmospheric N through symbiotic association with *Rhizobium* bacteria is the basis of their significance in agricultural systems, since increasing their use may decrease use of synthetic N fertilizers while maintaining acceptable production (Graham and Vance, 2003). *Fabaceae* are also able to acidify their rhizosphere and thus, mobilize P (Hinsinger et al., 2011). To increase benefits of using *Fabaceae* in agricultural systems, it is necessary to improve knowledge about links between their growth strategies and their adaptations to different levels of resource availability or environmental factors, i.e., ecological niches (Hutchinson, 1957).

Plants' functional traits (Violle et al., 2007) are generally used as proxies to determine species' growth strategies and ecological niches (Garnier, 1992; Grime et al., 1997; Díaz et al., 2004; McGill et al., 2006). At the biome scale, when functional trait syndromes of two species overlap, their growth strategies and ecological niches tend to be similar (Díaz et al., 2004; Wright et al., 2004; Westoby and Wright, 2006). As a result, comparative approaches developed in functional ecology have become increasingly used by agronomists (Garnier and Navas, 2011) to determine species' abilities to withstand stresses (Richardson et al., 2011; Comas et al., 2013) or to provide services (Ansquer et al., 2009; Damour et al., 2014). At the same time, technical progress has made lot of resolved phylogenies easily available for large ranges of species. Consequently phylogeny has begun to be used to study species coexistence rules (Webb, 2000). This approach is based on the hypothesis that phylogenetic relatedness among species could be used as a proxy of the overlap in their ecological niches (Cavender-Bares et al., 2004; Mayfield and Levine, 2010). Studies have also shown that functional traits carry both phylogenetic and ecological information (Webb et al., 2002; Cavender-Bares et al., 2009; Kunstler et al., 2012; Swenson, 2013). Should functional traits values be phylogenetically correlated, it could also be hypothesized that: (i) closely related species would have similar values for functional trait; (ii) the phylogenetic approach could complete the functional traits measurement particularly for difficult to measure traits, such as root traits. However, since these hypotheses were often proposed but rarely tested, the strength of the relations between functional traits, phylogenetic relatedness and ecological niches needs to be assessed (Cavender-Bares et al., 2004; Swenson and Enquist, 2009; Kraft and Ackerly, 2010). To that end we tested which distances between phylogenetic and functional traits distances are more closely related to species' ecological niches distances.

Hutchinson (1957) defines species' ecological niche as a hyper-volume in the multidimensional space of ecological variables within which a species can maintain a viable population. As a consequence, species' ecological niche distances can be defined as the distance between their positions along these ecological variables. To compare species' ecological niches, we estimated their niche positions along ecological variables based on ecological indicator values (Ellenberg et al., 1991; Pervanchon, 2004). Although some species have wide ranges of positions along environmental gradients, these indicators enable representing species optima along them (Wahl and Ryser, 2000), i.e., nutrient availability (N, P) and environmental constraints (pH, salinity, continentality). Consequently, differences in indicator values between species should be interpreted as their ecological niche distance.

As reported by Kunstler et al. (2012), there are two ways to express functional trait distances: absolute *|t_A_* – *t_B_|* or hierarchical (*t_A_* − *t_B_*) trait distance, where *t_A_* and *t_B_* are functional trait values of species *A* and *B*, respectively. If traits responsible for ecological niche differentiation are conserved in phylogeny, the most related species should have similar trait values, i.e., low absolute trait distance. They should also have similar values of ecological indicators, i.e., low absolute ecological indicator distance *|i_A_* – *i_B_|*, where *i_A_* and *i_B_* are ecological indicator values of species *A* and *B*, respectively. This ecological indicator distance should also be related to absolute trait distance. However, if there is high niche differentiation, traits should not be conserved in phylogeny, and closely related species may have greatly different trait values and ecological indicator values, i.e., high absolute trait and ecological indicator distances.

As for the absolute trait distance, if trait *t* is related to species' *A* and *B* niche differentiation the hierarchical trait distance (*t_A_* − *t_B_*) should be correlated with ecological indicator distance. The hierarchical trait distance appeared to be much more efficient than the absolute trait and the phylogenetic distances to explain the strength and the direction of species interaction among different growth conditions, using a few leaf (Kunstler et al., 2012) and root (Fort et al., 2014) functional traits. It is assumed that few leaf functional traits can describe species' strategies and position along environmental gradients (Díaz et al., 2004; McGill et al., 2006; Bernard-Verdier et al., 2012). Nevertheless, traits-based studies are limited by partial knowledge of pertinent functional traits that can explain species' ecological niche positions along environmental gradients. This is particularly true when considering root traits, which are likely to play a major role in plant adaptation to different ecological niches.

In this study, our main objective is to evaluate the relevance of phylogenetic distance, absolute trait distance and hierarchical trait distance in comparing species' adaptations to different habitats. We chose to work with wild grassland *Fabaceae* because we hypothesized that they would have wider ranges of growth strategies adapted to stressful conditions than species used in agricultural systems, which are selected for high fertility (Lynch, 2007). Among these species, we measured several functional traits (root, leaf, and whole-plant traits); we chose these traits according to their presumed or demonstrated relations with their growth strategies and ecological requirements (Westoby, 1998; Wahl and Ryser, 2000; Roumet et al., 2006; Poorter and Markesteijn, 2008; Mommer et al., 2011; Fort et al., 2015). Species' phylogenetic and ecological distances were assessed from species-level phylogeny and species' ecological indicator values [continentality, edaphic humidity, pH, nitrogen availability, salinity, phosphorus (P) availability], respectively. Using these phylogenetic, functional, and ecological distances, our objectives were (i) to evaluate to what extent ecological niche distances between *Fabaceae* species are related to their absolute trait distance, phylogenetic distance or hierarchical trait distance and (ii) to identify traits associated with the adaptation of *Fabaceae* species to contrasting habitats.

## Materials and methods

### Species and growth conditions

We selected 13 *Fabaceae* species (Table 1) according to their preferences for habitats with contrasting nutrient availabilities and climates (Ellenberg et al., 1991; Pervanchon, 2004). Seeds were collected from wild populations in southwestern France in locations representative of their habitat preferences.

**Table 1:** **Species studied, their Ellenberg indicators for climate continentality (C), soil water availability (HE), soil N availability (N), soil pH (pH), and soil salinity (S) (Ellenberg et al., 1991), P requirement (P) (Pervanchon, 2004)**.

**Species**	***C***	***HE***	***N***	***P***	**pH**	***S***
*Anthyllis vulneraria* L.	3	3	2	2.9	7	0
*Lotus corniculatus* L.	3	7	4	0.0	7	4
*Medicago lupulina* L.	NA	4	NA	3.8	8	0
*Medicago sativa* L.	7	3	NA	10.0	9	0
*Melilotus albus* Medik.	6	3	4	NA	7	0
*Onobrychis viciifolia* Scop.	6	3	3	4.0	8	0
*Securigera varia* L. (Lassen)	5	4	3	NA	9	0
*Trifolium campestre* Schreb.	3	4	3	4.6	6	0
*Trifolium fragiferum* L.	5	7	7	8.2	8	4
*Trifolium pratense* L.	3	5	NA	0	6	0
*Trifolium repens* L.	NA	5	6	0	6	1
*Vicia cracca* L.	NA	6	NA	3.8	NA	1
*Vicia tenuifolia* Roth.	6	3	2	8.3	8	0

*C, HE, N, and pH are expressed on a scale from 1 (oceanic habitats, low requirement for water and N, low soil pH requirement) to 9 (continental habitats, high requirements for water and N, high soil pH requirement). P is expressed on a scale from 0 (no particular P requirement) to 10 (high P requirement). S is expressed on a scale from 0 (very sensitive to salinity) to 9 (hyperhaline species)*.

NA indicates “not available.”

Three to five seeds were sown in pots 10 cm in diameter × 1 m deep, containing 10 kg (dry weight basis) of soil each. The substrate used was a 1:1 volume mixture of sand and calcareous clay soil with basic pH (8.3) and a high carbonate concentration (52.2 g.kg^−1^). The total N concentration measured in the substrate was 0.46 g N kg^−1^ and the organic C concentration was 3.72 g.kg^−1^. At the beginning of the experiment, 2 g of phosphorus (P) were added per pot in the form of commercial triple super phosphate (Eurofertil) to reach a total concentration of 1.51 g.kg^−1^ and to provide high P availability to the plants with a Olsen *P-*value (Olsen et al., 1954) equalling 54 mg P_2_O_5_kg^−1^. Pots were watered twice a day by micro-diffusers placed on the soil surface to ensure that soil moisture remained close to field capacity. Pots were arranged in a greenhouse to form 6 blocks, with one pot of each species randomly placed within each block. Two weeks after germination one plantlet was kept per pot; consequently, we grew 78 individuals over 130 days from 12 October 2011 to 20 February 2012. All pots were maintained at 20°C during the day and 17°C at night, and the day:night ratio was 16 h:8 h with a mean of 380 μmol m^2^ s^−1^ PAR during the day.

### Leaf traits

**Figure 1:**
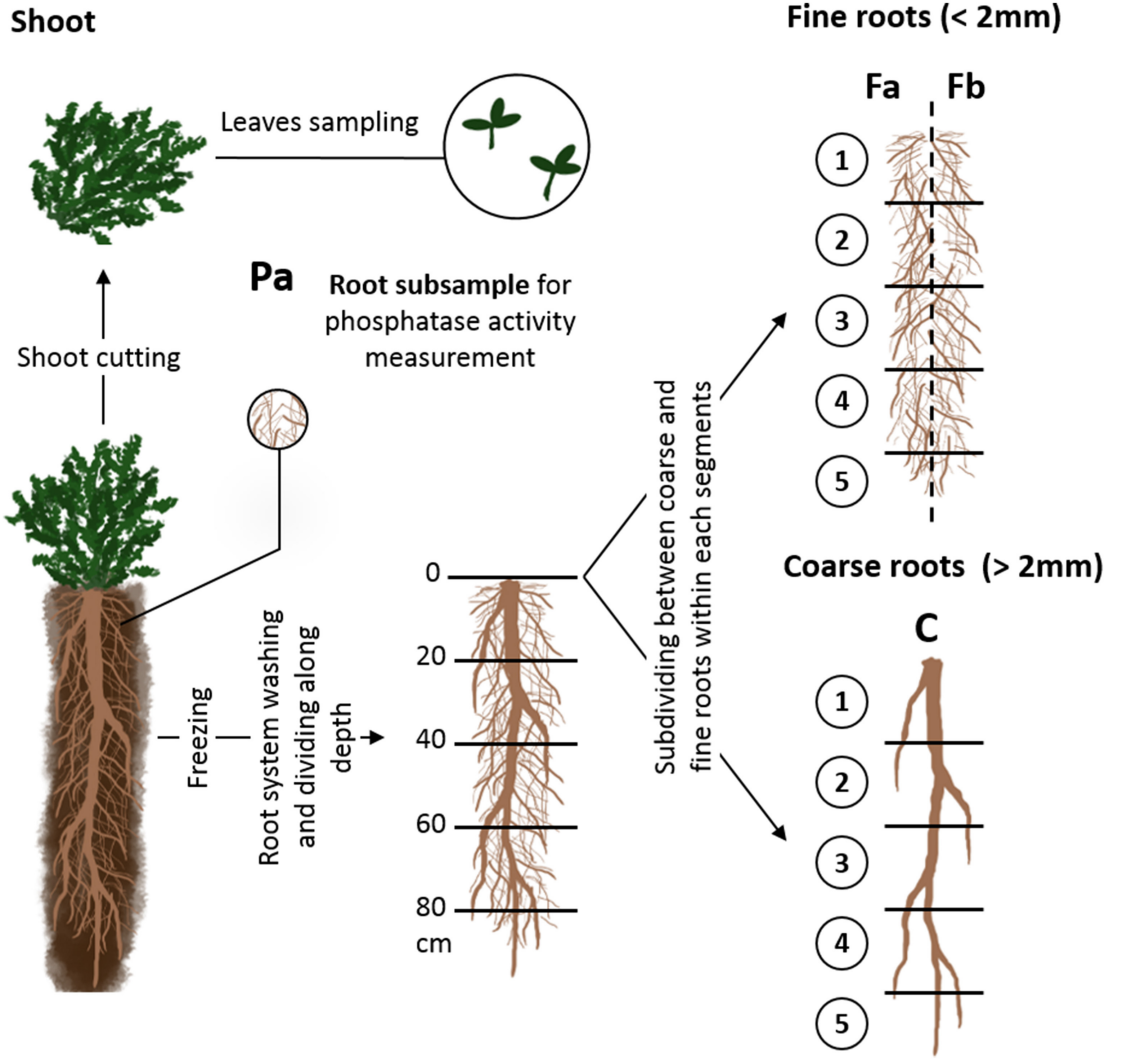
**Illustration of the successive steps of sampling during the experiment**. C: coarse root fraction; Fa and Fb two halves (fresh weight basis) of fine root fractions of each soil segments; Pa: root sample for phosphatase activity measurements.

During harvest (Figure 1), the aboveground vegetative portion of each individual was collected and stored for 6 h at 4°C in plastic boxes containing water to saturate plants. One and six young mature leaves of each individual were collected for species with large and small leaves, respectively, to ensure that the dry mass was high enough to be impacted little by measurement errors. Fresh leaves of each individual were weighed and then scanned and their surface area measured; leaves were then dried for 48 h at 60°C and weighed as the remaining aboveground biomass. Specific leaf area (SLA) was calculated as the ratio between leaf area and leaf dry mass, and leaf dry matter content (LDMC) was calculated as the ratio between leaf dry mass and leaf fresh mass.

### Root traits

After shoot clipping, pots were opened lengthwise and a subsample of fine roots (Figure 1 Pa) was removed to measure root-surface phosphatase activity as described by Fort et al. (2015). Afterwards, remaining roots were gentle washed under water and frozen (−18°C) to conserve them until functional traits measurements (Oliveira et al., 2000).

### Histologic measurements

Next, individual root systems were defrosted; four 1-cm root segments were removed from order-1 or -2 roots at least 2 cm above the apex. Root segments were prepared as described by Fort et al. (2015). For each individual, we determined the mean percentage of root cross-sectional area occupied by aerenchyma and by stele in the four root sections.

### Root measurements

After sampling for anatomical measurements, root systems of each individual were cut into five 20-cm-long segments carefully washed of remaining soil particles. Within each segment taproots (diameter >2 mm and with secondary growth) were separated from fine roots which were considered appropriate for measuring functional traits (<2 mm in diameter, Cornelissen et al., 2003). Next, within each segment, the fine root fraction was divided into two halves on the basis of sample fresh weight (fractions Fa and Fb, Figure 1). Fractions Fa 1 to Fa 5 (Figure 1) and the coarse root fractions of each segment, i.e., fractions C 1 to C 5 (Figure 1), were dried separately at least 48 h at 60°C and weighed to estimate the distribution of coarse and total root biomass with depth. Afterward, they were grinded and their P concentration was measured (van Veldhoven and Mannaerts, 1987).

The other half of fine root fractions (Fb 1 to Fb 5, Figure 1) of each plant were pooled in water, homogenized by agitation and half (i.e., 25% of each plant's total fine root biomass) was sampled and kept for mycorrhizal rate measurement. All the nodules of the remaining half of fine root fraction were removed, dried at least 48 h at 60°C and weighed. The fine roots and the taproot fractions, free of nodules, were stained and scanned using the same method used to measure root surface phosphatase activity. We used open-source Fiji software (Schindelin et al., 2012) to measure root hair length. For each individual, we measured the length of root hairs on 10 randomly distributed points of the root sample. After scanning, each root sample was drained, dried at least 48 h at 60°C and weighed.

### Mycorrhization rate measurement

To stain the arbuscular mycorrhizal fungi (AMF) within roots we used methods proposed by Vierheilig et al. (1998). Mycorrhization rates were quantified using the grid-lines intersect method (Giovannetti and Mosse, 1980). Stained roots were spread in a Petri dish marked with a grid on the bottom. At each root-gridline intersect, the presence or absence of AMF was noted and the mycorrhization rate was calculated as the ratio of the number of intersects with AFM to the total number of intersects counted (300 on average).

### Trait calculations

Mean root diameter (D), root tissues density (RTD), and specific root length (SRL) were measured on the nodule-free fine root sample. Mean root diameter (mm) was calculated as the mean of the median diameter of each root-diameter class (provided by WinrhizoTM Pro 2007) weighted by the root length in each class. The very fine root percentage was calculated as the percentage of root length with a diameter < 0.2 mm (Roumet et al., 2006). SRL (m.g^−1^) was calculated by dividing the sample root length by its dry mass. RTD (g.cm^−3^) was the ratio of the sample root dry mass to its volume (Cornelissen et al., 2003). Root phosphorus use efficiency (RPUE) was calculated by dividing the SRL by the root P concentration. Specific investment in nodules was calculated as the ratio of the nodule dry mass of a scanned root sample to its root mass.

The percentage of coarse root was calculated as coarse root weight divided by total root system weight. Specific taproot length (STRL) and taproot tissue density (TRTD) were calculated as the taproot sample length and volume, respectively, divided by the taproot sample dry mass. RLD (cm cm^−3^) was calculated as the ratio of the total fine root length (thin root dry mass divided by SRL) plus the taproot length within the pot to the total volume of the pot. To analyse root distribution along the profile, we used the depth of 95% of the fine root length, calculated using a linear regression of cumulative fine root length with depth.

### Statistical analyses

We built a phylogenetic tree of the 13 species from their plastid matK gene (Wojciechowski et al., 2004) using the neighbor-joining method. This tree was consistent with the general tree of the *Fabaceae* family of Wojciechowski et al. (2004). To determine the influence of phylogeny on species' trait values, variance analysis of species trait values was performed using eigenvectors associated with the phylogenetic tree (Diniz-Filho et al., 1998). From this tree, we calculated a phylogenetic distance matrix. Among species, we also calculated pair-wise absolute and hierarchical distance matrices for each trait and ecological indicator.

To use the same procedure to test whether phylogenetic or ecological niche distances were related to functional trait differences among species, we used a series of Mantel correlation tests (Legendre and Legendre, 2012). This test compares the observed Mantel's statistic (Mantel's *r*) to the Mantel's *r* calculated from a random distribution generated from 999 permutations of the distance matrix's rows and columns. A more positive correlation than expected by chance between species phylogenetic or ecological indicator and functional trait absolute distances would indicate that the more species are phylogenetically or ecologically distant, the more their functional trait attributes differ.

Using the same Mantel test, we tested whether hierarchical trait distances correlated with hierarchical ecological indicator distances. A significant correlation (more positive or negative than expected by chance) between these two hierarchical distances would indicate that functional trait values tend to vary along ecological gradients. A positive correlation between hierarchical distance matrices of ecological indicators and functional traits would indicate that species with higher ecological indicator values have higher trait attributes than other species. In contrast, a negative correlation would indicate that species with high ecological indicator values have lower trait values than other species. Statistical analyses were performed with R 2.15.1 software. Significant differences were determined at α = 0.05.

## Results

Large differences existed among species' attributes for nearly all functional traits and biomass production (Table 2), highlighting their wide range of strategies for resource acquisition and management. Among fine root traits, the least variable trait was root diameter. Fine root biomass, RPUE, taproot biomass and the root:shoot ratio were the most variable traits, with coefficients of variation higher than 100% (Table 2). Fine root functional traits, whole root system traits (except for depth of 95% of root length) and taproot traits all displayed high variability among *Fabaceae* species, whereas aboveground traits and total biomass had the lowest coefficients of variation.

**Table 2:** **Functional trait minimum, mean, maximum and coefficient of variation values of 13 *Fabaceae* species (*n* = 13)**.

**Functional traits**	**Minimum**	**Mean**	**Maximum**	**Coefficient of variation (%)**
**FINE ROOT TRAITS (<2 mm)**				
Aerenchyma (%)	0.0	5.18	12.9	68.2
Diameter (mm)	0.13	0.25	0.37	29.2
Hairs (μm)	7.9	19.8	39.6	49.8
Investment in nodules (g.m^−1^)	0.02	0.13	0.34	71.4
Mycorrhizal rate (%)	0.8	10.4	26.0	75.9
Root phosphorus use efficiency (m.mg^−1^)	20.5	97.5	424.4	107.3
Root-surface phosphatase activity (μg.m^−1^.h^−1^)	140	378.3	740	48.4
Root tissue density (mg.cm^−3^)	54.9	96.4	209.6	43.5
Specific root area (dm^2^.g^−1^)	4.8	17.3	34.5	45.2
Specific root length (m.g^−1^)	38.0	215.9	655.6	72.7
Stele percentage (%)	9.3	17.5	38.0	47.3
Very fine root percentage (<0.2 mm)	15.0	51.4	91.0	48.2
**TAPROOT ROOT TRAITS**				
Specific taproot length (m.g^−1^)	0.24	1.30	2.24	54.9
Taproot tissue density (g.cm^−3^)	1.61	2.9	5.35	36.6
**WHOLE ROOT SYSTEM TRAITS**				
Depth of 95% root length (cm)	81.8	88.4	99.7	5.3
Fine root biomass (g)	0.42	2.53	10.55	106.2
Root system biomass (g)	1.43	5.17	14.81	85.1
Root length density (cm.cm^−3^)	1.23	4.26	9.94	48.9
Taproot biomass (g)	0.25	2.62	13.53	156.9
Taproot percentage (%)	7.4	41.1	91.8	73.7
**ABOVEGROUND TRAITS**				
Specific leaf area (m^2^.kg^−1^)	19.2	24.4	31.1	17.4
Leaf dry matter content (mg.g^−1^)	138	202.7	288	22.2
Aboveground biomass (g)	4.0	13.3	19.07	30.1
**WHOLE PLANTS TRAITS**				
Root:shoot ratio	0.07	0.50	1.9	109.2
Total biomass (g)	8.4	18.5	27.8	26.9

*Functional trait values of the 13 Fabaceae species are presented in the Supplementary Material*.

**Figure 2:**
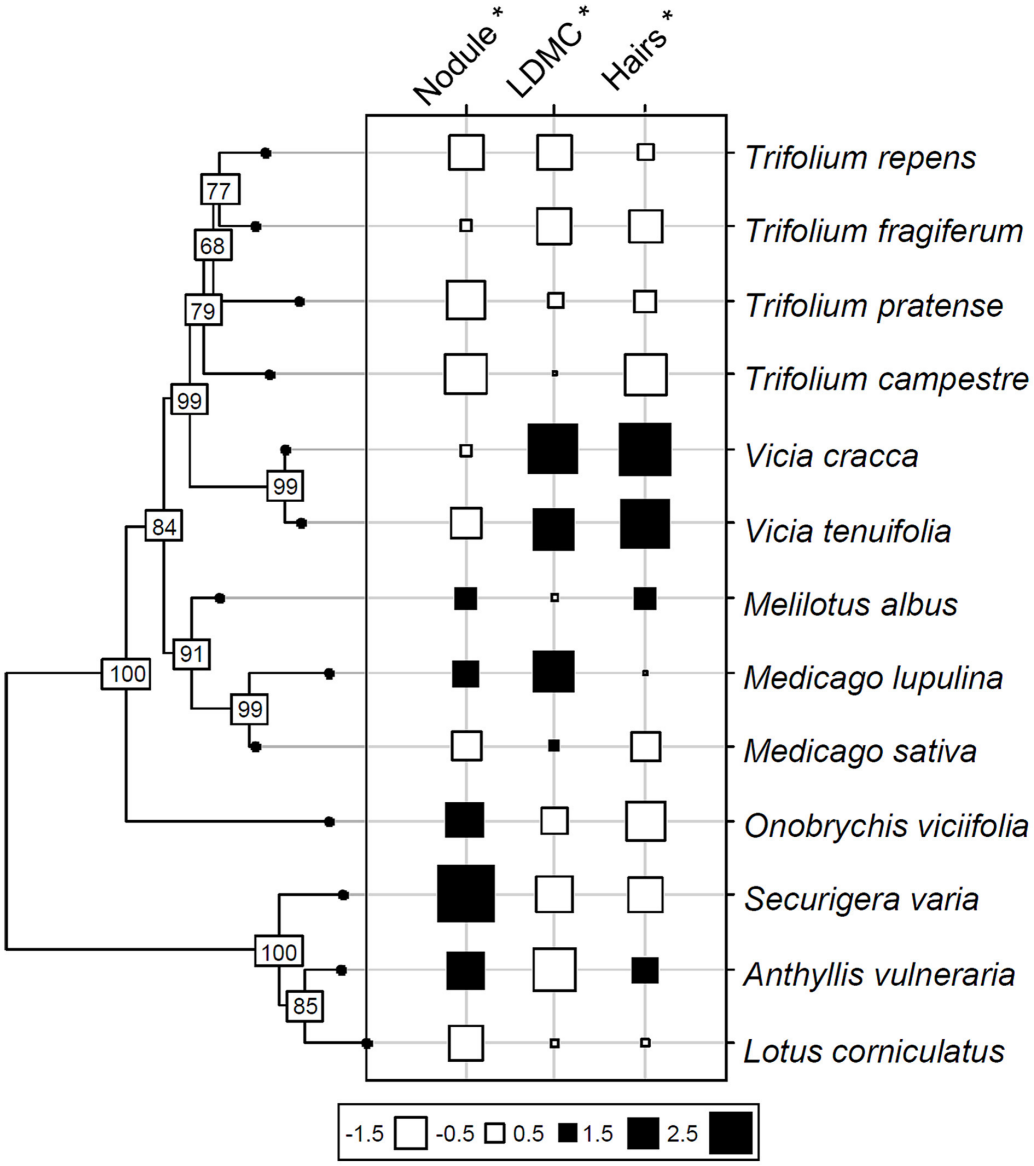
**Phylogeny of 13 grassland *Fabaceae* species based on published sequences of the matK gene (Wojciechowski et al., 2004)**. Values of three functional traits significantly related to phylogeny are shown at the tips of the phylogeny. Trait values are normalized; values on axis nodes represent bootstrap results. Black squares: high trait values; white squares: low trait values. Nodule biomass per unit of root length (Nodule), leaf dry matter content (LDMC) and root hair length (Hairs) were the traits carrying a significant phylogenetic signal.

Based on variance analysis, only three functional traits had values significantly correlated with species phylogeny: specific investment in nodules, root hair length and LDMC (Table 3, Figure 2). The significant relation of nodule investment was due to a difference between the closely related *Trifolium* and *Vicia*, which had lower nodule biomass per root length than the other species (except *Medicago sativa* and *Lotus corniculatus*). Significant correlations of root hair length and LDMC with phylogeny were due to *Trifolium* spp. having lower trait values than *Vicia* spp., *Melilotus albus* and *Medicago lupulina* (Table 3, Figure 2).

**Table 3:** **Results of variance analysis of the phylogenetic signal among functional traits and results of the Mantel test (*r-* and *p*-values) between pair-wise species absolute trait distances and phylogenetic distance and absolute ecological indicator distance**.

**Absolute trait distances**	**Phylogenetic signal**	**Phylogenetic distance**	**Absolute ecological indicator distance**		
			***C***	***P***	***p_H_***
	***F*-value**	**Mantel's r**	**Mantel's r**		
**FINE ROOTS (<2 mm)**					
Aerenchyma		0.52**			
Hairs	4.12*				
Investment in nodules	3.98*	0.32*			
**WHOLE ROOT SYSTEM**					
Depth of 95% of root length				0.37*	0.65**
Root system biomass			0.33*	0.40*	
Taproot proportion				0.48*	
**ABOVEGROUND SYSTEM**					
Leaf dry matter content	5.21*				
**WHOLE PLANT**					
Root:shoot ratio				0.43*	

Absolute trait distances not shown in this table did not have significant relations with phylogeny or at least one absolute ecological indicator distance (^*^p < 0.05, ^**^p < 0.01). Ecological indicators: C, continentality; P, phosphorus availability; pH, soil alkalinity.

### Phylogenetic and ecological absolute distances

**Figure 3:**
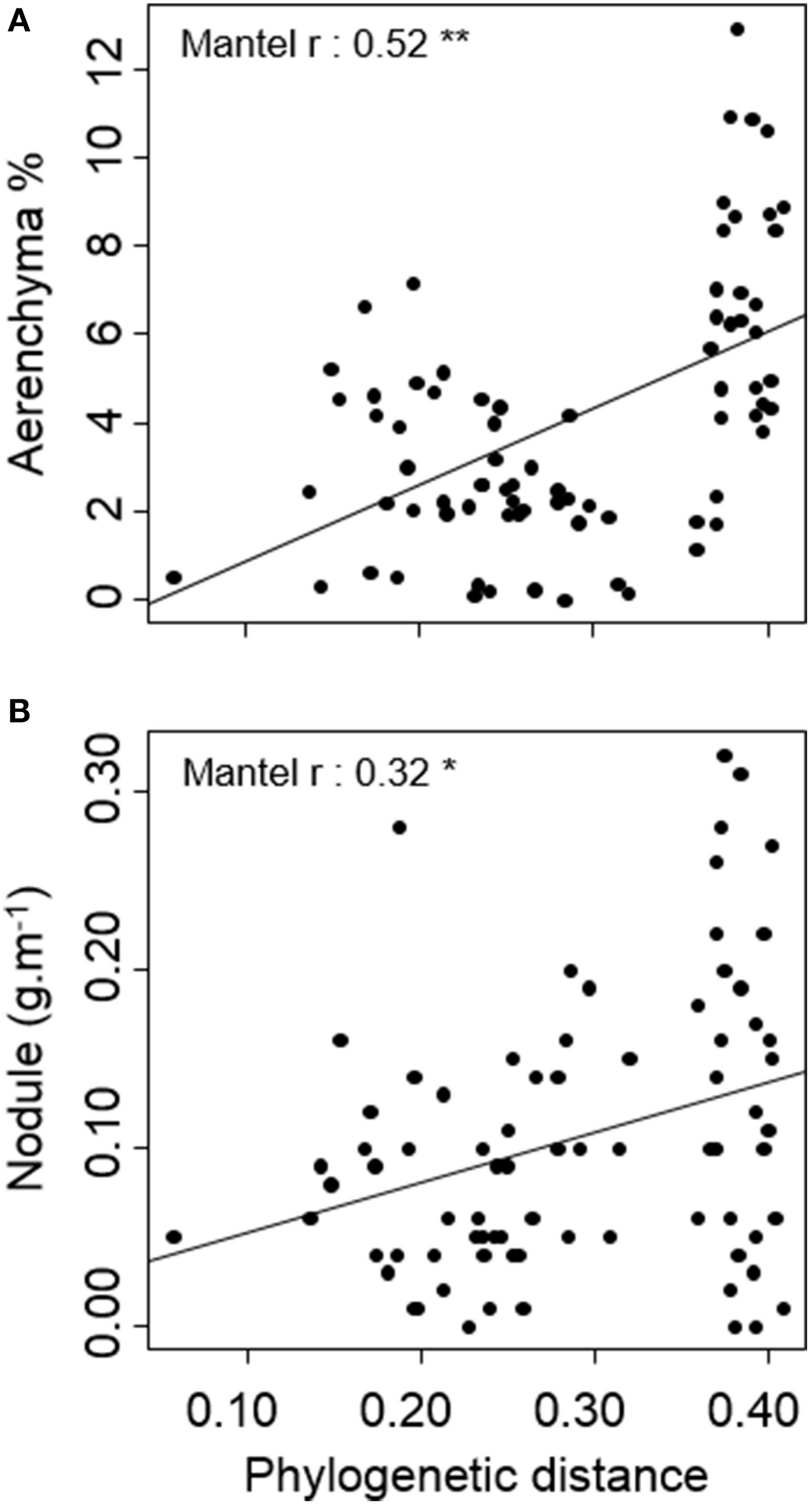
**Relation between phylogenetic distance and pair-wise species' (A) aerenchyma (% of root cross-sectional area occupied by aerenchyma) and (B) nodule (nodule biomass per root length) absolute distances**. Lines represent linear regressions between pair-wise species traits and phylogenetic distance. ^*^p < 0.05, ^**^p < 0.01.

Among the 25 traits studied. Only two showed differences among species related to phylogenetic distance: cross-sectional area occupied by aerenchyma and, as in the variance analysis, specific investment in nodules (Table 3, Figure 3). Ecological indicators' absolute distances did not correlate significantly with phylogenetic distances, showing that species' ecological distances were not related to their phylogenetic distances.

**Figure 4:**
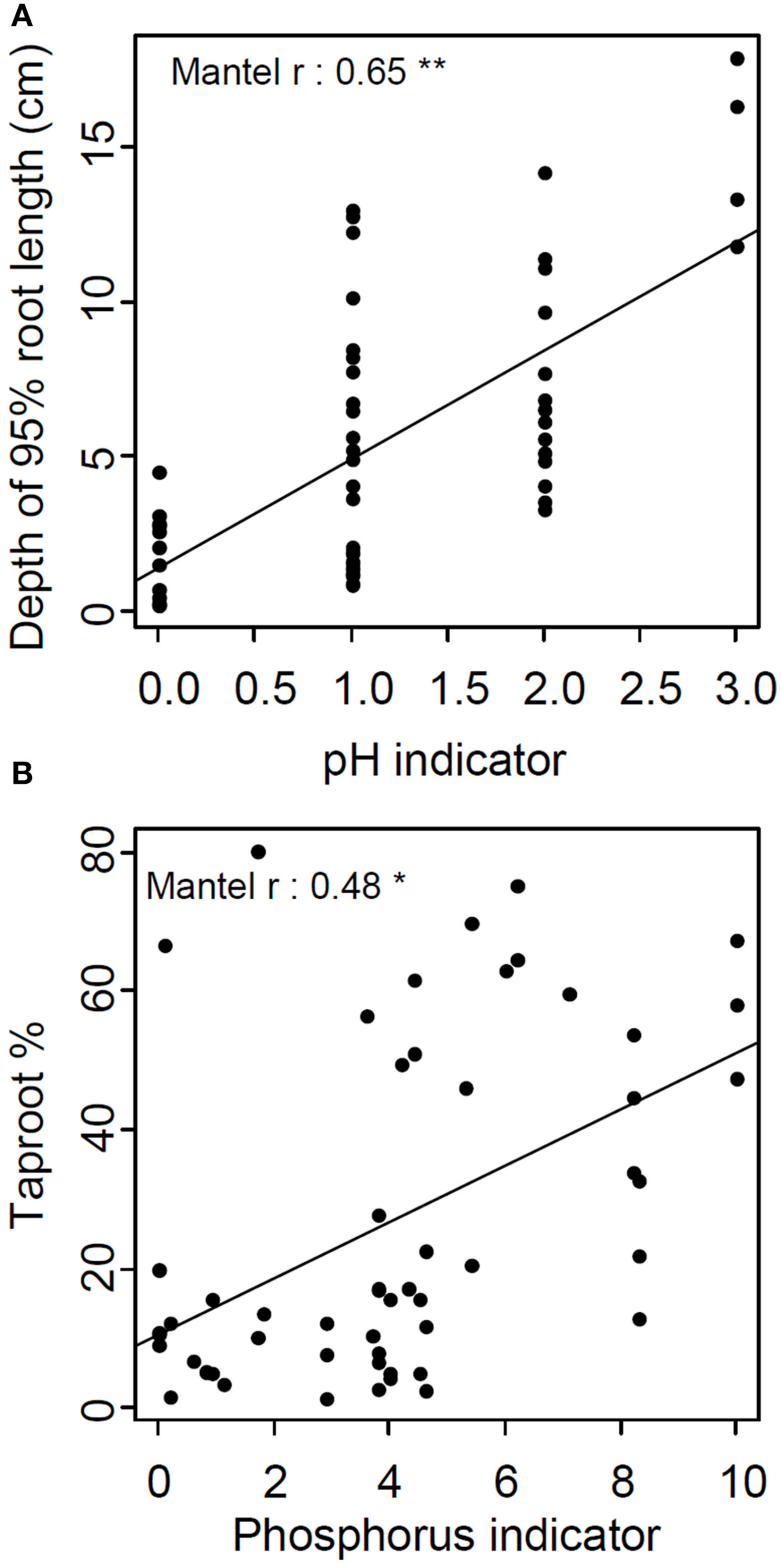
**Relation between absolute pair-wise species' distances of (A) pH indicator and depth of 95% of root length (B) and phosphorus indicator and taproot percentage**. Lines represent linear regressions between pair-wise species traits and ecological indicator distances (^*^p < 0.05, ^**^p < 0.01).

Continentality and pH indicators' absolute distances were significantly correlated with root system biomass and depth of 95% of root length, respectively. The P indicator's absolute distance was the only one correlated with four traits: depth of 95% of root length, root system biomass, taproot % and root:shoot ratio (Table 3, Figure 4). In all cases, Mantel's *r*-values were positive, highlighting larger absolute trait distances between phylogenetically or ecologically distant species than between closely related species. It is interesting to note that traits carrying phylogenetic signals were not those correlated with ecological indicators.

### Hierarchical distances

Hierarchical traits and ecological distances appeared to be closely related (Table 4). Aboveground biomass, root hair length, and RTD hierarchical trait distances were the only ones not related to ecological indicator hierarchical distances. Traits most related to ecological indicators were taproot traits, whole-root system traits, and whole-plant traits, which correlated on average with three, three and four ecological indicators, respectively.

**Table 4:** Results of Mantel test (*r*- and *p*-values) between pair-wise species hierarchical trait distances and hierarchical ecological indicator distances (^*^p < 0.05, ^**^p < 0.01, ^***^p < 0.001).

**Hierarchical trait distances**	**Hierarchical ecological indicator distance**					
	***C***	***HE***	***N***	***P***	**pH**	***S***
**FINE ROOT (<2 mm)**						
Aerenchyma (%)		0.53***				0.64***
Diameter (mm)	0.53**					
Hairs (μm)						
Investment in nodules (g.m^−1^)					0.48**	
Mycorrhizal rate (%)		0.52***	0.42*			0.43**
Root phosphorus use efficiency (m.mg^−1^)	−0.54***					
Root-surface phosphatase activity (μg.m^−1^.h^−1^)	0.55**	−0.34**				
Root tissue density (mg.cm^−3^)						
Specific root length (m.g^−1^)	−0.50**				−0.44*	
Specific root surface (dm^2^.g^−1^)	−0.46*				−0.44**	
Stele percentage (%)	−0.51**				−0.42**	
Very fine root percentage (<0.2 mm)	−0.60***				−0.35*	
**TAPROOT ROOT**						
Specific taproot length (m.g^−1^)	−0.51**		−0.37*	−0.36*		
Taproot tissue density (g.cm^−3^)		−0.28*	−0.55***			−0.31**
**ROOT SYSTEM**						
Depth of 95% of root length (cm)	0.55**			0.70***	0.85***	
Fine root biomass (g)			−0.59**			
Biomass (g)	0.71***	−0.44***		0.58***		
Root length density(cm.cm^−3^)	−0.55**		−0.44*		−0.62***	−0.31*
Taproot biomass (g)	0.62**	−0.34**		0.61***		
Taproot proportion (%)	0.52*		0.45*	0.54***	0.44**	
**ABOVEGROUND SYSTEM**						
Specific leaf area (m^2^.kg^−1^)				0.47**		
Leaf dry matter content (mg.g^−1^)						
Biomass (g)						
**WHOLE PLANTS**						
Root:shoot ratio	0.63***	−0.37**		0.62***	0.29*	
Biomass (g)	0.48*	−0.48***		0.34*		−0.28*

*Ecological indicators: C, continentality; HE, edaphic humidity; N, nitrogen availability; P, phosphorus availability; pH, soil alkalinity; S, salinity*.

**Figure 5:**
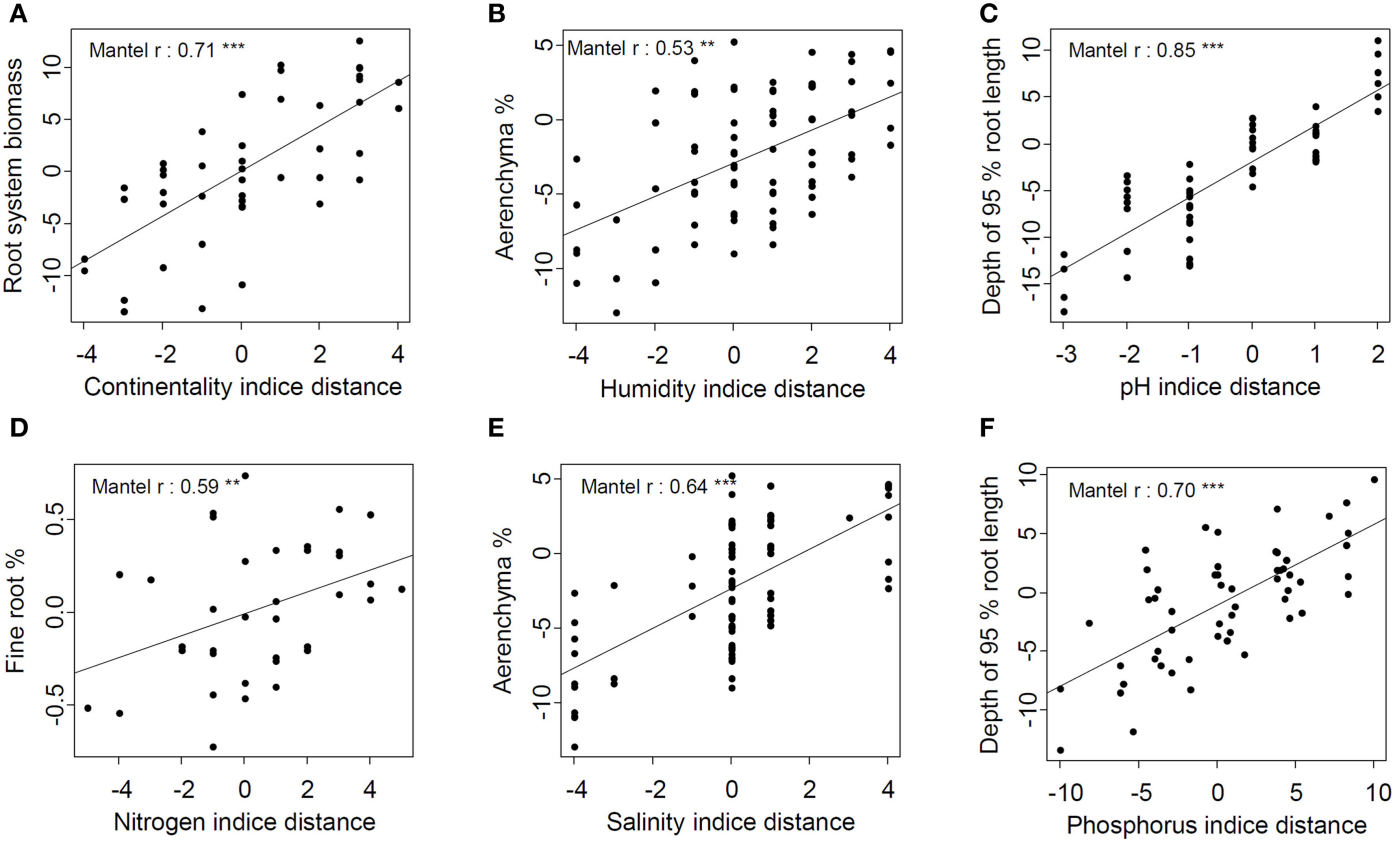
**Best relations between hierarchical trait distance and each ecological indicator's hierarchical distance**. **(A)** Root system biomass and continentality indicator; **(B)** Aerenchyma % and humidity indicator; **(C)** Depth of 95 % root length and pH indicator; **(D)** Fine root % and nitrogen indicator; **(E)** Aérenchyma % and salinity indicator; **(F)** Depth of 95 % root length and Phosphorus indicator. Lines represent linear regressions between pair-wise species traits and ecological indicator distances (^**^p < 0.01, ^***^p < 0.001).

The continentality indicator hierarchical distance correlated with 14 traits hierarchical distances, highlighting that species from continental habitats had thicker root and taproot (lower SRL and associated traits), higher root biomass, investment in taproot, root:shoot ratio, deeper root systems, and total biomass than species from oceanic habitat (Table 4). Among all traits, root system biomass hierarchical distance appeared to be the most related to continentality indicator hierarchical distance (Figure 5A). Likewise, edaphic humidity indicator hierarchical distance correlated with eight hierarchical trait distances, highlighting that species from wet habitats had higher root cross-sectional area occupied by aerenchyma, higher mycorhizal rates, lower TRTD and biomass, lower root biomass, lower total biomass, and lower root:shoot ratio than species from dry habitats (Table 4). Aerenchyma hierarchical distance was the most related to edaphic humidity indicator hierarchical distance (Figure 5B).

As for relations between the pH indicator and traits, Mantel's correlations highlighted that species from habitats with alkaline soil had higher investment in taproot nodule biomass and root:shoot ratio, deeper root systems, thicker roots and lower RLD than those from habitats with acidic soil (Table 4). The depth of 95% of root length hierarchical distance was the trait most correlated with pH indicator and P indicator hierarchical distances (Figures 5C,F). Hierarchical P indicator distance was the only one which did not correlate with fine-root traits but did correlate with SLA. Species from habitats with high P availability had higher rooting depth, root system and taproot biomass, taproot percentage, SLA, root:shoot ratio, and total biomass, but lower STRL than species from habitats with low P availability. Surprisingly, RPUE and root surface phosphatase activity, traits that are supposed to be closely related to P acquisition, were not related to the P indicator.

N indicator and salinity indicator hierarchical distances were less related to hierarchical trait distances. Species from eutrophic habitats had higher mycorrhizal rates and taproot percentage but lower STRL, TRTD, fine-root biomass, and RLD than species from oligotrophic habitats. Among all hierarchical trait distances, fine-root biomass was the most related to the N indicator (Figure 5D). Species from saline habitats had higher cross-sectional root area occupied by aerenchyma and mycorrhizal rates than species from non-saline habitats but lower taproot density, RLD, and total biomass. As for the edaphic humidity indicator, the aerenchyma hierarchical distance was most correlated with the salinity hierarchical distance (Figure 5E).

## Discussion

### Relations among traits, phylogeny, and ecological indicator distances

We demonstrated that differences in ecological niches between grassland *Fabaceae* species were linked more to species' trait differences than to their phylogenetic relatedness. The lack of significant relation between ecological indicator distances and phylogenetic distances showed that adaptation to an ecological constraint was not the prerogative of one lineage. Our results challenge the purpose of using phylogeny as a proxy for functional traits and to describe ecological differences (Webb, 2000; Cavender-Bares et al., 2009) among closely related species from the *Fabaceae* family.

It is interesting to note that *Trifolium* and *Vicia* species differ the most in their trait attributes, even though they are closely related genera (Wojciechowski et al., 2004), highlighting low inheritance of trait values from the deep node of the phylogeny but strong conservatism between closely related taxa (within genera). This result could explain the lack of correlation previously observed between functional trait attributes and phylogenetic relatedness in a subtropical forest (Uriarte et al., 2010). As in previous studies on competition (Kunstler et al., 2012; Fort et al., 2014), absolute trait distances appear to be a poor predictor of species ecological niche distances, while hierarchical trait and hierarchical ecological indicator distances were highly correlated. The more species differ in their ecological niches, i.e., have different values of ecological indicators, the more they differ in trait values. As a result, grassland *Fabaceae* functional traits tend to converge among species having the same ecological requirements, i.e., which are likely to coexist (Mayfield and Levine, 2010). Results confirm that absolute trait distance is statistically inferior to hierarchical trait distance for predicting species' ecological niches, as previously demonstrated for species interactions (Kunstler et al., 2012; Fort et al., 2014). This highlights the interest in considering hierarchical trait distance in comparative plant studies.

### Trait and ecological indicator hierarchical distances

For grassland *Fabaceae*, root traits were linked more to species ecological demands than leaf traits. This may have been related to fact that root traits varied more than leaf traits among the species considered. We showed that, for *Fabaceae*, adaptation to a continental climate results in root systems characterized by more conservative strategies than those of species adapted to an oceanic climate, which have acquisitive strategies. Conservative species have root-trait syndromes linked to low nutrient-acquisition efficiency, e.g., high mean diameter, low SRL, low fine root percentage, but high resource-conservation capacity, e.g., high root system and taproot biomasses, high root:shoot ratio. In contrast, acquisitive species have root-trait syndromes linked to highly efficient nutrient-acquisition strategies, e.g., high fine-root percentage, RLD, SRL, and low root:shoot ratio (Craine et al., 2002; Tjoelker et al., 2005; Roumet et al., 2006; Picon-Cochard et al., 2011; Fort et al., 2014). This trend is confirmed by the fact that species adapted to low edaphic humidity, a characteristic of continental habitats (Ejrnaes and Bruun, 2000), had high TRTD and biomass, root system biomass and root:shoot ratio. Thus, species with acquisitive growth strategies are well-represented in habitats with low stress (e.g., drought, nutrient shortage) and an oceanic climate (e.g., steady temperature and precipitation). In contrast, conservative species are mainly found in stressful continental climates (with hot, dry summers, and cold winters). As a result, for grassland *Fabaceae*, this study establishes a strong link between the root economic spectrum, i.e., resource acquisition vs. conservation trade-off at the root system level and species' abilities to compete in non-stressful meadows or to tolerate stress (Grime, 1977).

This ability to withstand stressful conditions could be linked to deep rooting, which allows plants to acquire water from deep soil horizons (Ho et al., 2005). Although growth conditions in pots may alter root distribution with soil depth, we showed that this root-trait syndrome allowing deep soil foraging was also linked to a high requirement for soil P availability. This trade-off between water acquisition efficiency linked to a deep root system and efficiency in acquiring P was previously observed in maize lineages (Ho et al., 2005). This can be explained by drought-tolerant species developing more root biomass in deep soil layers, which generally have lower available P (Hinsinger, 2001) than surface horizons, where available P tends to be higher (Richardson et al., 2011). The correlation observed between pH and rooting depth agrees with previous results of pH tolerance of grassland legumes. For example, Zahran (1999) reported that *M. sativa* was much more sensitive to soil acidity than *Lotus* species (*L. tenuis* Waldst. and Kit); similarly, we observed that *M. sativa* had a deeper root system than *L. corniculatus*. This correlation showed that deep-rooted *Fabaceae* species are more common in alkaline soils. One can hypothesize that acid toxicity in deep layers due to Al toxicity explains the shallow rooting depth of species adapted to acid soil. Moreover, P mobility is low in dry and alkaline soils (Hinsinger, 2001), decreasing its availability, which may explain why these species need habitats with high P fertility.

Mycorrhizal rate hierarchical distances correlated with edaphic humidity indicator hierarchical distances, highlighting that species with high values for these functional traits under non-limiting water and P supplies are also those adapted to habitats with high water supply. This can be explained by the behavior of AMF in dry conditions that increases the efficiency of water extraction: by delaying stomata closure, the host plant can extract water at a matrix potential below wilting point (Jung et al., 2012). During long and dry periods, however, this strategy can be harmful for highly mycorrhized plants by depleting soil water, decreasing their survival chances under dry growth conditions. This is consistent with the fact that high investment in AMF symbiosis is a disadvantage in dry environments (Zangaro et al., 2008). Mycorrhizal rate hierarchical distance also correlated positively with the N indicator, which reinforces the idea that acquisitive species, from N-rich habitats, have high mycorrhizal rates. Therefore, we hypothesize that investing in AMF symbiosis to acquire resources is not a useful strategy under highly stressful conditions, as reported by Lambers et al. (2008) for P stress.

However, the positive link observed between the S indicator and mycorrhizal rates confirms that AMF helped species tolerate salinity (Jung et al., 2012). Cross-sectional area occupied by aerenchyma hierarchical distance was positively related with HE and S indicator hierarchical distances, showing that high aerenchyma production helps species withstand stresses (Richardson et al., 2011). This also highlights that aerenchyma allows species to grow in wet habitats by ensuring oxygen transport in waterlogged soil (Hodge et al., 2009).

## Conclusion

Results demonstrate that ecological niche distances measured among grassland *Fabaceae* were strongly related to their root hierarchical trait distances, whereas phylogenetic and absolute trait distances appeared to be poor predictors of their ecological distances. These results highlight the interest in characterizing root functions to rethink the choice of species sown in agricultural systems according to their capacity to grow efficiently within sub-optimal growth conditions. This functional approach may be suitable for differentiating, within a species, genotypes adapted to low fertility or drought tolerance. However, further work is needed to test whether these relations between hierarchical trait distances and ecological niches could be extended to and within other plant families.

### Conflict of interest statement

The authors declare that the research was conducted in the absence of any commercial or financial relationships that could be construed as a potential conflict of interest.
