# Supplementary material for: Hierarchical traits distances explain grassland Fabaceae species' ecological niches distances
Source: Front Plant Sci. 2015 Feb 17;6:63. doi: 10.3389/fpls.2015.00063 (PMC4330681; doi:10.3389/fpls.2015.00063)
Supplement: Supplementary file 1 [file Table1.DOCX]

**Table 1.** Functional trait values of 13 *Fabaceae* species (mean ± SE, n=6), within lines different letters indicate significant differences (ANOVA)

| **Functional traits** | **Species** | | | | | | | | | | | | |
| --- | --- | --- | --- | --- | --- | --- | --- | --- | --- | --- | --- | --- | --- |
|  | *Anthyllis vulneraria* | *Lotus corniculatus* | *Medicago lupulina* | *Medicago sativa* | *Melilotus albus* | *Onobrychis viciifolia* | *Securigera varia* | *Trifolium campestre* | *Trifolium fragiferum* | *Trifolium pratense* | *Trifolium repens* | *Vicia cracca* | *Vicia tenuifolia* |
| **Fine root traits (<2mm)** |  |  |  |  |  |  |  |  |  |  |  |  |  |
| Aerenchyma (%) | 8.4 ab | 12.9 a | 2.3 cde | 2 de | 7.2 bc | 4.2 bcde | 9 ab | 0 e | 6.6 bcd | 2 de | 4.2 bcde | 4.55 bcde | 4 bcde |
| Diameter (mm) | 0.25 c | 0.28 b | 0.18 e | 0.25 c | 0.29 b | 0.36 a | 0.22 d | 0.13 f | 0.2 de | 0.21 d | 0.19 de | 0.37 a | 0.34 a |
| Hairs (µm) | 25.6 b | 19.4 bcd | 19.7 bcd | 14.2 def | 23.9 bc | 9.3 ef | 12.2 def | 7.9 f | 12.9 def | 17 cde | 18.5 bcd | 39.6 a | 38.1 a |
| Investment in nodosity | 0.22 bc | 0.06 ef | 0.17 bcd | 0.08 def | 0.16 bcd | 0.22 b | 0.34 a | 0.02 f | 0.12 cde | 0.03 ef | 0.06 ef | 0.12 def | 0.07 ef |
| Mycorrhizal rate (%) | 7.4 def | 8.6 de | 0.9 f | 5.2 ef | 0.8 f | 5.5 ef | 23.4 ab | 17.9 bc | 26 a | 8.7 de | 11.2 cde | 14.3 cd | 5.8 ef |
| Root phosphorus use efficiency (m.mg-1) | 77.2 bcd | 61.7 cd | 76.9 bcd | 70.7 bcd | 46 cd | 22.7 d | 93.9 bc | 424.4 a | 95.4 bc | 129 b | 126.9 b | 22.6 d | 20.5 d |
| Root-surface phosphatase activity (µg.m-1.h-1) | 293 cde | 402 c | 175 ef | 354 c | 740 a | 600 b | 210 def | 140 f | 304 cd | 369 c | 323 cd | 350 c | 658 ab |
| Root tissue density (mg.cm-3) | 54.9 h | 110.8 c | 83 defg | 68.2 fgh | 55.7 gh | 94.4 cde | 90.6 cde | 75.6 efg | 96.2 cd | 77.9 defg | 87 def | 149.1 b | 209.6 a |
| Specific root area (dm2.g-1) | 23.8 b | 11.1 d | 21.7 bc | 19.7 bc | 18.2 c | 10.3 de | 17.2 c | 34.5 a | 17.3 c | 20.3 bc | 18.9 c | 6.7 ef | 4.8 f |
| Specific root length (m.g-1) | 248.5 bc | 110.2 ef | 306.7 b | 210 cd | 171 de | 80.3 fg | 211.1 cd | 655.6 a | 224.9 cd | 252.1 bc | 247.7 bc | 50.6 fg | 38 g |
| Stele percentage (%) | 17.4 de | 13.4 de | 27.6 b | 12.8 de | 11.3 f | 15.3 de | 15.9 de | 38 a | 18.4 cd | 13 de | 26 bc | 9.3 ef | 9.7 def |
| Very fine root percentage (<0.2mm) | 57 e | 39 fg | 75 b | 44 f | 37 g | 17 h | 63 de | 91 a | 70 bc | 68 cd | 73 bc | 15 h | 19 h |
| **Taproot root traits** |  |  |  |  |  |  |  |  |  |  |  |  |  |
| Specific taproot length (m.g-1) | 2.24 a | 1.55 cd | 1.14 de | 0.24 f | 0.24 f | 2.19 ab | 0.4 f | 1.28 de | 1.24 de | 1.62 bcd | 0.77 ef | 2.06 abc | 1.5 cd |
| Taproot tissue density (g.cm-3) | 3.8 bc | 2.66 def | 4.06 b | 1.61 g | 1.86 fg | 5.35 a | 2.14 efg | 4.02 b | 1.95 fg | 3.05 cd | 2.16 efg | 3.26 bcd | 2.88 de |
| **Whole root system traits** |  |  |  |  |  |  |  |  |  |  |  |  |  |
| Depth of 95% root length (cm) | 88.6 c | 85.5 cd | 86.7 cd | 95.1 ab | 88.3 c | 87.4 cd | 99.7 a | 83.4 cd | 89.5 bc | 87 cd | 81.8 d | 88.9 bc | 86.9 cd |
| Fine root biomass (g) | 1.56 de | 3.25 c | 1.09 de | 1.37 de | 1.27 de | 3.05 c | 1.04 de | 1.17 de | 0.42 e | 1.55 de | 1.71 d | 4.85 b | 10.55 a |
| Root system biomass (g) | 2.18 e | 4.72 c | 1.44 e | 10.88 b | 14.81 a | 3.98 cd | 4.45 c | 1.43 e | 1.7 e | 2.54 de | 2.17 e | 5.47 c | 11.48 b |
| Root length density | 4.83 bcd | 4.53 bcd | 4.28 bcd | 3.72 bcd | 2.91 de | 3.09 cde | 2.66 de | 9.94 a | 1.23 e | 4.83 bcd | 5.41 b | 2.85 de | 5.12 bc |
| Taproot biomass (g) | 0.61 def | 1.44 d | 0.33 ef | 9.49 b | 13.53 a | 0.91 def | 3.39 c | 0.25 f | 1.26 de | 0.97 def | 0.45 ef | 0.57 def | 0.84 def |
| Taproot percentage (%) | 28 d | 29.4 d | 22.9 de | 87.6 a | 91.8 a | 24.6 de | 77.34 b | 17.8 ef | 74.1 b | 40.2 c | 20.3 e | 12.4 fg | 7.4 g |
| **Aboveground traits** |  |  |  |  |  |  |  |  |  |  |  |  |  |
| Specific leaf area | 21 ef | 26.1 cd | 25.6 cd | 29.9 ab | 21.3 ef | 19.6 f | 30.6 ab | 31.1 a | 23.4 cde | 20 ef | 22.9 def | 19.2 f | 26.8 bc |
| Leaf dry matter content (mg.g-1) | 138 f | 202 c | 268 ab | 206 c | 202 c | 184 cd | 158 ef | 203 c | 157 ef | 196 cd | 173 de | 288 a | 260 b |
| Aboveground biomass (g) | 17.27 ab | 11.92 cde | 19.07 a | 11.15 de | 9.01 e | 15.33 abc | 4 f | 11.63 cde | 12.07 cde | 13.86 bcd | 17.03 ab | 14.64 bcd | 16.28 ab |
| **Whole plants traits** |  |  |  |  |  |  |  |  |  |  |  |  |  |
| Root:shoot ratio | 0.12 de | 0.39 d | 0.07 e | 1 bc | 1.9 a | 0.26 de | 1.17 b | 0.12 de | 0.14 de | 0.18 de | 0.13 de | 0.35 d | 0.73 c |
| Total biomass (g) | 19.5 bc | 16.6 cd | 20.5 bc | 22 b | 23.8 ab | 19.3 bc | 8.4 e | 13.1 de | 13.8 de | 16.4 cd | 19.2 bc | 20.1 bc | 27.8 a |
